# Can We Improve Stavudine's Safety Profile in Children? Pharmacokinetics of Intracellular Stavudine Triphosphate with Reduced Dosing

**DOI:** 10.1128/AAC.00761-18

**Published:** 2018-10-24

**Authors:** Steve Innes, Louvina van der Laan, Peter L. Anderson, Mark Cotton, Paolo Denti

**Affiliations:** a Family Infectious Diseases Clinical Research Unit, Department of Paediatrics and Child Health, Faculty of Medicine and Health Sciences, Stellenbosch University, Cape Town, South Africa; b Desmond Tutu TB Centre, Department of Paediatrics and Child Health, Faculty of Medicine and Health Sciences, Stellenbosch University, Cape Town, South Africa; c Division of Clinical Pharmacology, Department of Medicine, University of Cape Town, Cape Town, South Africa; d Department of Pharmaceutical Sciences, University of Colorado Denver, Anschutz Medical Campus, Aurora, Colorado, USA

**Keywords:** antiretroviral agents, children, intracellular drug concentration, population pharmacokinetics, stavudine

## Abstract

Stavudine remains a useful replacement option for treatment for HIV^+^ children. WHO reduced the adult dose to 30 mg twice daily, which maintains efficacy and lowers mitochondrial toxicity. We explored intracellular stavudine triphosphate levels in children receiving a reduced dose of 0.5 to 0.75 mg/kg of body weight twice daily to investigate whether a similar dose optimization can safely be made. A population pharmacokinetic model was developed to describe the pharmacokinetics of intracellular stavudine triphosphate in 23 HIV^+^ children and 24 HIV^+^ adults who received stavudine at 0.5 mg/kg and 20 mg twice daily for 7 days, respectively. Simulations were employed to optimize the pediatric dosing regimen to match exposures in adults receiving the current WHO-recommended dose of 30 mg twice daily. A biphasic disposition model with first-order appearance and disappearance described the pharmacokinetics of stavudine triphosphate. The use of allometric scaling with fat-free mass characterized well the pharmacokinetics in both adults and children, and no other significant effect could be detected. Simulations of 30 mg twice daily in adults predicted median (interquartile range [IQR]) stavudine triphosphate minimum drug concentration (*C*_min_) and maximum drug concentration (*C*_max_) values of 13 (10 to 19) and 45 (38 to 53) fmol/10^6^ cells, respectively. Targeting this exposure, simulations in HIV^+^ children were used to identify a suitable weight-band dosing approach (0.5 to 0.75 mg/kg), which was predicted to achieve median (IQR) *C*_min_ and *C*_max_ values of 13 (9 to 18) and 49 (40 to 58) fmol/10^6^ cells, respectively. Weight-band dosing using a stavudine dose of 0.5 to 0.75 mg/kg is proposed, and it shows comparable exposures to adults receiving the current WHO-recommended dose of 30 mg twice daily. Our pharmacokinetic results suggest that the decreased stavudine dose in children >2 years would have a reduced toxic effect while retaining antiretroviral efficacy.

## Abstract (precis)

Stavudine remains a useful replacement option for treatment for HIV^+^ children. WHO reduced the adult dose to 30 mg twice daily, which maintains efficacy and lowers mitochondrial toxicity.

## INTRODUCTION

In 2016, the United Nations Children's Fund (UNICEF) estimated that 2.1 million children under 15 years of age were living with HIV, the majority in sub-Saharan Africa (1). Following a failed first-line regimen, second- and third-line drug options for children in sub-Saharan Africa are limited and have a greater risk of drug-related adverse events (2). Laboratory monitoring for drug-related toxicities is unreliable in many parts of sub-Saharan Africa, and the ideal second- and third-line drug options need to have robust short-term safety with potent antiretroviral efficacy to attain full virological suppression, whereafter children can be switched to less effective but safe drug options, such as abacavir. The cost of second- and third-line antiretroviral drugs is high (3), and cost-effective approaches in resource-limited settings are important.

Stavudine is a nucleoside reverse transcriptase inhibitor with potent activity against HIV. It is being phased out as a first-line antiretroviral treatment option due to slow cumulative mitochondrial toxicity but remains an important replacement option for treatment for HIV-positive children. At the recommended dose, it causes lipoatrophy in up to one-third of patients (4–6), although this effect emerges only after 9 to 18 months of exposure (7). Lipoatrophy is a fat distribution disorder that can cause the limbs and face to become disfigured, leading to stigmatization and reduced adherence to therapy (8). The risk of developing lipoatrophy in patients on stavudine is dose dependent (9–11), with cumulative mitochondrial toxicity driven especially by peak drug concentration (*C*_max_). In 2007, an influential review of the previous 15 years' data by Hill et al. (9) showed that a lower stavudine dose of 20 mg for adults <60 kg and 30 mg for adults >60 kg twice daily results in a markedly lower risk of lipoatrophy while maintaining antiviral efficacy. This led the World Health Organization to recommend a reduction in the adult dose from 40 mg to 30 mg twice daily irrespective of weight (12). The dose for children, however, has not yet been reduced. Consequently, children on stavudine continue to be administered a disproportionately high dose, which may result in more rapid accumulation of metabolic adverse effects than in adults on the reduced dose.

The current standard pediatric dose of stavudine (1 mg/kg of body weight twice daily) was determined through linear extrapolation from the pharmacokinetic parameters of the adult dose of 40 mg twice daily (13, 14). Dose finding studies showed that an oral dose of 1 mg/kg twice daily in children under 30 kg results in plasma exposure similar to that of an adult over 60 kg taking 40 mg twice daily, and that an oral dose of 0.5 mg/kg twice daily in children results in plasma exposure similar to that of an adult over 60 kg taking 20 mg twice daily (13, 14).

Stavudine has rapid absorption and good oral bioavailability and is not protein bound. The clearance of stavudine is minimally affected by hepatic metabolism, as it is eliminated unchanged via the kidneys (15). The antiviral and toxic effects of stavudine are dependent on the intracellular concentration of its phosphorylated metabolite, stavudine triphosphate (16). Becher et al. described a linear relationship between intracellular stavudine triphosphate and plasma stavudine samples, inferring that a measurement from either would be clinically useful (17).

We compared intracellular stavudine triphosphate levels in children receiving a reduced dose to adults receiving 20 or 30 mg twice daily. We used a population pharmacokinetic model to describe the pharmacokinetics of stavudine triphosphate in HIV-infected adults and children. This technique provides a semimechanistic platform to interpret pharmacokinetic data and is able to account for the concomitant effect of multiple factors, such as weight and age. Population pharmacokinetics of stavudine and stavudine triphosphate in adults with model-predicted stavudine triphosphate concentrations in children have been described in previous published reports (18, 19). These previous studies were used to inform our model for intracellular stavudine triphosphate in children.

## RESULTS

### Patients and data description.

Twenty-three HIV-positive children and 24 HIV-positive adults were included in an established antiretroviral therapy regimen, contributing to a total of 188 samples. All the recruited participants were virally suppressed with adequate adherence, except for one child whose samples were excluded following nonadherence to the trial drug. Two of the stavudine triphosphate samples were below the lower limit of quantitation (LLOQ).

A summary of the participants' characteristics is presented in Table 1.

**TABLE 1:** Summary of patient characteristics in HIV-infected children and adults receiving stavudine^*a*^

Characteristic	Adults (*n* = 24)	Children (*n* = 23)
Age (yr)	36 (30–40) (26–51)	8 (7–9) (4–11)
No. (%) of males	1 (4)	12 (52)
Wt (kg)	83 (70–98) (62–158)	23 (20–26) (18–31)
Fat-free mass (kg)	47 (44–51) (39–77)	19 (16–20) (14–25)
Body mass index (kg/m^2^)	31 (26–40) (24–55)	16 (14–17) (13–19)
CD4 count (10^6^ cells/liter)	586 (473–835) (389–1,263)	947 (822–1,507) (281–2,951)
Serum creatinine (μmol/liter)^*b*^	53 (49–56) (41–70)	29 (26–30) (20–39)

a Data are presented as the median (interquartile range) (range), unless otherwise stated.

b Lab reference is 49 to 90 μmol/liter for adults and 30 to 48 μmol/liter for children.

### Pharmacokinetic model.

**FIG 1:**
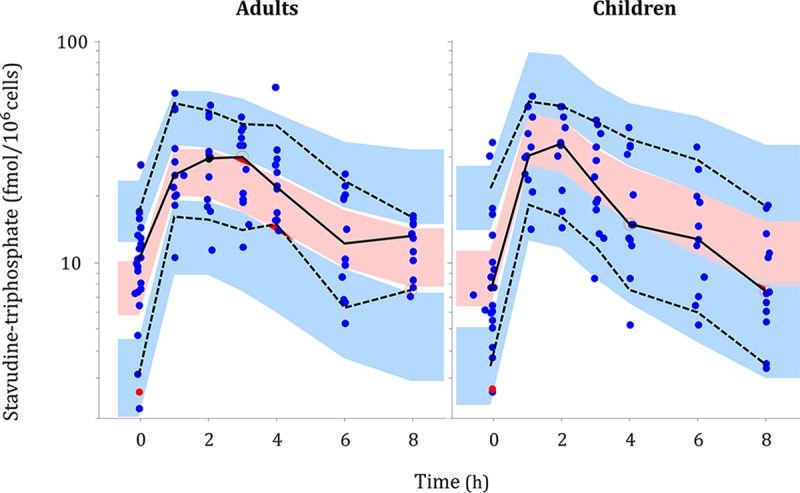
Visual predictive check of the pharmacokinetic model for stavudine triphosphate stratified by children versus adults, using 1,000 simulations. Observed data are displayed as filled circles, including censored data points (below the LLOQ) in red. The solid and dashed lines represent the 10th, 50th, and 90th percentiles of the observed data, while the shaded areas (pink and blue) are the model-predicted 90% confidence intervals for the same percentiles.

A biphasic disposition model with first-order appearance and disappearance suitably described the pharmacokinetics of stavudine triphosphate in peripheral blood mononuclear cells (PBMC). The use of a biphasic as opposed to monophasic model improved the model fit significantly (24 point decrease in −2 log-likelihood [−2LL], 2 degrees of freedom, *P* < 0.01). The final parameter estimates are presented in Table 2. A visual predictive check (VPC) plot stratified by adults and children is displayed in Fig. 1, showing that the 10th, 50th, and 90th percentiles of the observed data are in agreement with the respective 90% confidence intervals simulated by the model, thus supporting the adequacy of the model.

**TABLE 2:** Parameter estimates of the final model for stavudine triphosphate in HIV-infected adults and children

Parameter^*a*^	Adult (%RSE^*b*^) (*n* = 24)	Child (*n* = 23)
CL (10^12^ cells/h)	454 (9)	230
*V*_c_ (10^12^ cells)	2,569 (18)	1,037
Q (10^12^ cells/h)	469 (30)	238
*V*_p_ (10^12^ cells)	7,500 (58)	3,027
*k*_a_ (1/h)	1.17^*c*^ (46)	
*F*	1 (fixed)	
Additive error (fmol/10^6^ cells)	0.6 (fixed)^*d*^	
Proportional error (%)	27.4 (8)	
IIV CL (%CV)	30.4 (21)	
IOV *F* (%CV)	36.2 (20)	
IOV *k*_a_ (%CV)	30.6 (61)	

a All clearance and volume of distribution parameters were estimated by allometric scaling using fat-free mass, and the typical values reported here refer to an adult with a median fat-free mass of 47.2 kg. The children values are also reported here for comparison, but they were not estimated separately in the model; they were just obtained by rescaling the adult values to the median fat-free mass of our children cohort, 19.1 kg. IIV and IOV were assumed to be log-normally distributed, and their magnitudes are reported here as approximate coefficient of variation (%CV). CL, apparent oral clearance; *V*_c_, apparent central volume of distribution; Q, apparent oral intercompartmental clearance; *V*_p_, apparent peripheral volume of distribution; *k*_a_, appearance rate constant; *F*, bioavailability, IIV, interindividual variability; IOV, interoccasion variability.

b RSE, relative standard error.

c A Bayesian prior with values from Horton et al. (18) was used for the estimation of *k*_a_.

d The estimate of the additive error was small and not stable, so it was fixed to 20% of the median LLOQ.

Interindividual variability (IIV) was supported on clearance and interoccasion variability (IOV) on bioavailability and the appearance rate constant (*k*_a_) for stavudine triphosphate. The estimate of the typical value of *k*_a_ for stavudine triphosphate was obtained using a weakly informative Bayesian prior based on a value reported in a population pharmacokinetic model for stavudine by Horton et al. (∼2.38 1/h) and 30% uncertainty (18). The prior was added to stabilize the model and avoid flip-flop kinetics (20). The final parameter values obtained when including the prior were similar to the ones without the prior. The estimate of additive error for stavudine triphosphate was not stable and was therefore conservatively fixed to 20% of the median LLOQ (3 fmol/10^6^ cells; range, 3 to 3.47 fmol/10^6^ cells) for stavudine triphosphate.

The inclusion of allometric scaling to account for size differences using fat-free mass provided a meaningful benefit in terms of model fit (13 point decrease in −2LL) and was therefore retained in the final model. The typical clearance for stavudine triphosphate for a child with a fat-free mass (FFM) of 19.1 kg was 230 × 10^12^ cells/h, and for an adult with a FFM of 47.2 kg, it was 454 × 10^12^ cells/h. Individual concentration-versus-time profiles (see Fig. S1 in the supplemental material) and compartmental pharmacokinetics (Table S1) for children and adults are presented in the supplemental material.

No effect of age (maturation), sex, or renal function could be detected. No significant differences were detected for bioavailability, clearance, or *k*_a_ of stavudine triphosphate between adults and children. The VPC for stavudine triphosphate stratified by children versus adults, shown in Fig. 1, shows that a model using only allometric scaling was suitable to describe the pharmacokinetics in both children and adults; the observed data fell within the confidence intervals predicted by the model for both groups. There was a trend toward slightly lower bioavailability for the children, but this did not reach statistical significance and did not provide convincing improvements to the diagnostic plots and VPCs.

### Dosing optimization.

The covariate sets for our *in silico* children and adult cohorts are summarized in Table S1.

**FIG 2:**
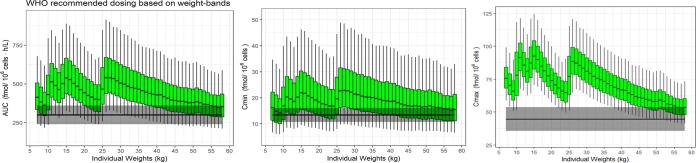
Simulated stavudine triphosphate exposure versus weight resulting from the WHO-recommended weight-band dosing of 1 mg/kg twice daily. The gray band represents the median simulated adult value ±20% for a stavudine dose of 30 mg twice daily.

Current WHO-recommended weight-band dosing for children results in dramatically higher intracellular drug exposure (up to double the *C*_max_) than that with the corresponding adult dose (Fig. 2). The model was used to simulate adult exposures with a 30-mg twice-daily dose and predicted median (IQR) minimum drug concentration (*C*_min_) and *C*_max_ values of 13 (10, 19) and 45 (38, 53) fmol/10^6^ cells, respectively, and median (IQR) area under the concentration-time curve (AUC) values of 300 (238, 380) fmol/10^6^ cells · h/liter.

**FIG 3:**
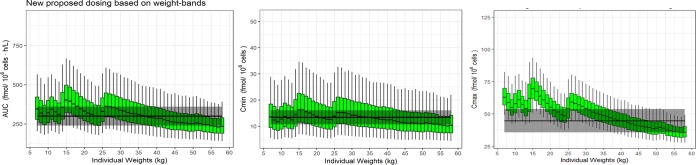
Simulated stavudine triphosphate exposure versus weight using the new proposed dosing guideline of 0.5 to 0.75 mg/kg twice daily. The gray band represents the median simulated adult value ±20% for a stavudine dose of 30 mg twice daily.

These values were used as reference ±20% when optimizing the dose in children. Simulations were used to devise a reduced-dose weight-band approach using 0.5 to 0.75 mg/kg twice daily. The newly proposed weight bands and dosing recommendations are presented in Table 3, and the simulated AUC, *C*_min_, and *C*_max_ values for stavudine triphosphate expected from these doses are presented in Fig. 3. The simulated values for *C*_min_ were based on a 12-h dosing schedule. Simulations predicted median (IQR) *C*_min_ and *C*_max_ of 13 (9, 18) and 49 (40, 58) fmol/10^6^ cells and a median (IQR) AUC of 300 (238, 379) fmol/10^6^ cells · h/liter in children.

**TABLE 3:** Stavudine dosing guidelines shown by number of capsules or milliliters by weight band twice daily for children >2 years

Body wt (kg)	WHO guideline, target 1 mg/kg twice daily	New proposed guideline, target 0.5–0.75 mg/kg twice daily^*a*^
7–9.9 (>2 years old)	9 ml (9 mg)^*b*^	7.5 ml (7.5 mg) or open 15-mg capsule into 5 ml water; give 2.5 ml^*b*^
10–13.9	1 capsule (15 mg)	10 ml (10 mg) or open 20-mg capsule into 5 ml water; give 2.5 ml^*b*^
14–16.9	1 capsule (20 mg)	1 (15 mg), 15 ml or open 15-mg capsule into 5 ml water^*b*^
17–19.9	1 capsule (20 mg)	1 (15 mg), 15 ml or open 15-mg capsule into 5 ml water^*b*^
20–24.9	1 capsule (20 mg)	1 capsule (15 mg)
25–29.9	1 capsule (30 mg)	1 capsule (20 mg)
30–59.9	1 capsule (30 mg)	1 capsule (20 mg)
>60	1 capsule (30 mg)	1 capsule (30 mg)

a Doses were rounded off to fall into a stavudine dose range of 0.5 to 0.75 mg/kg twice daily based on the current available capsules (15, 20, and 30 mg) and liquid formulation (1 mg/ml) strengths.

b The proprietary liquid formulation requires refrigeration. For patients who do not have access to a refrigerator, an adult capsule may be opened and dispersed in water.

Simulations predict that children dosed according to new weight-band dosing (range, 0.5 to 0.75 mg/kg) twice daily achieve median exposures within 20% of those in adults receiving a 30-mg twice-daily stavudine dose. The median *C*_max_ for children receiving the reduced stavudine dose was marginally higher than that for adults, especially for the smaller children.

## DISCUSSION

This is the first study to describe the pharmacokinetics of intracellular stavudine triphosphate in children. A biphasic model provided the best structural fit and may be a reflection of the two-compartmental kinetics of stavudine in plasma, as described before (18).

Allometric scaling suitably accounted for the effect of body size on the pharmacokinetic parameters of stavudine triphosphate in children receiving the reduced stavudine dose of 0.5 mg/kg twice daily and adults receiving 20 mg twice daily. Other than the effect of body size, no other significant differences in the pharmacokinetics could be detected between adults and children. The trend observed for a lower bioavailability in children might be due to the different formulations used in adults and children or to the difficulty in drug administration in children. A slightly lower bioavailability for children has been described for stavudine (13), but our study size limited further evaluation.

Simulations for children receiving a reduced stavudine weight-band dose (range, 0.5 to 0.75 mg/kg) twice daily achieved favorable *C*_min_ and AUC values of stavudine triphosphate compared to simulated median target adult ranges receiving 30 mg twice daily, which is the WHO dosing recommendation for adults. For children weighing more than 50 kg, the median exposures are at the lower range of the adult target value. Since the review by Hill et al. (9) showed that a lower stavudine dose of 20 mg twice daily for adults <60 kg maintains antiviral efficacy, and the current adult target in our simulations is based on a 30-mg twice-daily dose, the exposures are still considered adequate. *C*_max_ values for children receiving the reduced dose were only marginally higher than those in adults, suggesting a comparable decreased toxic effect. Exposures of children receiving the current weight-band WHO-recommended dose of 1 mg/kg were markedly higher than those with the target adult value, thus raising concerns of an increased toxic effect.

The simulated *C*_min_ in children in our study for a 0.5 mg/kg dose was lower (10.9 versus 14.8 fmol/10^6^ cells), and the *C*_max_ was higher (41.4 versus 23.9 fmol/10^6^ cells) than those reported in a similar study (19); however, simulations in the previous study were based on a model only informed by adult data.

For adults receiving 30 mg twice daily, our simulated median steady-state concentration of 25 fmol/10^6^ cells was lower, as previously reported by Becher et al. (37 fmol/10^6^ cells) (17). This was a study of 13 adults from a population (*n* = 28) with advanced disease (46% classed as Centers for Disease Control and Prevention stage C; unsuppressed viral load; mean CD4 count, 134 × 10^6^ cells/liter; mean weight, 54 kg at baseline), which might change the percentage of activated cells (i.e., lymphocytes) leading to a change in phosphorylation.

Our study had limitations. We did not analyze the plasma stavudine concentrations, which could have enriched the model by informing on the transition between stavudine (extracellular) and stavudine triphosphate (intracellular). On the other hand, stavudine and stavudine triphosphate levels are strongly correlated (17), so while our model could not separate the phenomena of stavudine absorption, penetration into cells, and conversion to stavudine triphosphate, it did describe suitably the intracellular concentrations of the active compound responsible for the drug efficacy (and toxicity), so we believe it is suitable for dose optimization. Even though the adult subjects were mainly female patients (96%), we do not expect any major effect of the gender distributions on the study findings. In the paper by Domingo et al. (16), no significant differences were found in the exposures of stavudine triphosphate (d4T-TP) between men and woman. Pregnancy was an exclusion criterion and was confirmed using serum β-human chorionic gonadotropin at screening. None of the participants reported taking oral or intramuscular injectable contraceptives; however, those on the three-monthly injectable contraceptive may not have thought to report it as a concomitant medication. Since stavudine does not affect liver enzymes and has minimal liver metabolism, it is unlikely to cause drug-drug interactions with hormonal contraceptives. Stavudine is mostly eliminated unchanged in urine (15). No effect of age could be detected, arguably because no children younger than 2 years were included in the study. Since we expect maturation to play an important role in this age range, stavudine triphosphate would need to be directly evaluated in separate studies. Finally, since we had a limited sample size (*n* = 47), further prospective studies would be needed to evaluate the pharmacokinetics, safety, and efficacy of the hypothesized optimized dosing regimen.

In conclusion, a stavudine dose of 0.5 mg/kg twice daily in children would result in exposures similar to those of adults receiving a 20-mg twice-daily dose. Weight-band dosing using a stavudine dose of 0.5 to 0.75 mg/kg is proposed, as it shows exposures comparable to those of adults receiving the current WHO-recommended dose of 30 mg twice daily. Our pharmacokinetic results suggest that a decreased stavudine dose in children (more than 2 years) would have a reduced toxic effect while retaining antiretroviral efficacy. Larger prospective studies would need to follow to confirm our findings.

## MATERIALS AND METHODS

### Study population.

The study was conducted in Cape Town, South Africa. The trial enrolled 24 prepubertal children (3 to 11 years) and 24 adults on standard local first-line antiretroviral therapy (specifically efavirenz, lamivudine, and abacavir for children, or tenofovir for adults) with a fully suppressed viral load for >12 months. Children had to be prepubertal (Tanner stage 1 or 2), as puberty may influence intracellular drug metabolism. The Tanner stage was determined by a pediatrician. Exclusion criteria were pregnancy, an acute medically significant event in the previous 6 months, and any grade 3 or greater laboratory screening investigations as defined by the International Maternal Pediatric Adolescent AIDS Clinical Trials (IMPAACT) network Pediatric/Maternal Diagnosis Appendix (Appendix 40 [http://www.fstrf.org/appendix40]). Patients on any of the following drugs known (or theoretically able) to cause drug-drug interactions with intracellular stavudine triphosphate were excluded: lopinavir, ritonavir, zidovudine, didanosine, doxorubicin, ribavirin, chloramphenicol, hydralazine, cisplatin, dapsone, ethambutol, ethionamide, hydralazine, isoniazid, lithium, metronidazole, nitrofurantoin, phenytoin, vincristine, and zalcitabine. Written informed consent was obtained. This pharmacokinetic trial was approved by the Health Research Ethics Committee of Stellenbosch University (trial reference M11/11/050) and by the Medicines Control Council of South Africa (trial reference 20111018).

### Study procedures.

Demographic and clinical characteristics were recorded, including WHO HIV clinical stage and anthropometrics. Concomitant medication and concurrent illnesses were sought by interview and physical examination. At screening, the following safety blood tests were performed: HIV viral load, CD4 count, complete blood count, cell differential, platelet count, and serum urea, creatinine, alanine transaminase, aspartate transaminase, alkaline phosphatase, total bilirubin, amylase, serum β-human chorionic gonadotropin levels (for females of childbearing potential). The preexisting effective antiretroviral treatment (ART) regimen for each participant was continued unaltered.

### Pharmacokinetic design.

This study used the proprietary pediatric liquid and capsule formulations of stavudine (Zerit; Bristol-Myers Squibb) for children and adults, respectively. Participants received a stavudine dose of 0.5 mg/kg twice daily for children or 20 mg twice daily for adults for 7 days. Adherence to trial drug was monitored by daily telephone calls and confirmed by pharmacy pill count.

Pharmacokinetic data were collected using sparse pharmacokinetic sampling on day 7. Blood samples were collected at 4 time points, at predose and either 1, 2, and 6 h or 3, 4, and 8 h after dosing. The sampling schedule was empirically chosen to provide an adequate representation of the absorption, elimination phase, *C*_max_, and *C*_min_. With the intention of describing the whole pharmacokinetic course while keeping the protocol less invasive, especially for children, it was decided to limit the maximum number of samples and use two alternative sampling schedules, with the patients randomized to either.

### Analytical method.

Peripheral blood mononuclear cells (PBMC) were extracted, and intracellular stavudine triphosphate concentrations were measured by liquid chromatography-tandem mass spectrometry. The assay method for the isolation of d4T-TP is analogous to previous assay methods (21). Briefly, 70:30 PBMC lysate is applied to Waters QMA strong anion exchange solid-phase extraction (SPE) column. A KCl salt gradient is utilized to isolate the TP fraction. This fraction was enzymatically dephosphorylated to d4T and applied to a Phenomenex Strata-X SPE cartridge for desalting and concentration of the sample. The detection of d4T and its respective stable labeled internal standard was accomplished on a Thermo Scientific TSQ Vantage triple quadrupole mass spectrometer coupled with a Waters Acquity ultraperformance liquid chromatography (UPLC) binary solvent manager and sample manager, utilizing positive electrospray ionization to detect the analytes. The assay range was 25.0 to 2,000 fmol/sample and was fit to a linear (1/concentration) weighted calibration curve. The accuracy and precision of the assay method were determined with quality controls. The interassay accuracy for the LLOQ (25.0 fmol/sample) was −3.0% and was within ±4.6% for the low quality control (QL; 75.0 fmol/sample), medium quality control (QM; 150 fmol/sample), and high quality control (QH; 1,500 fmol/sample). The interassay precision was 17.7% for the LLOQ and ≤10.3% for the other levels. During sample analysis, additional LLOQ samples were included for both calibration standards and quality controls in case resulting subject data were detectable but below the 25.0 fmol/sample LLOQ. These additional levels included 10.0, 15.0, and 25.0 fmol/sample concentrations. The samples were run in triplicate, and 67% of each level was needed to pass acceptance to be included in the analysis. The LLOQ for stavudine triphosphate depended on the assay run LLOQ and the estimated number of cells assayed for each patient and ranged from 3 to 3.47 fmol/10^6^ cells.

### Population pharmacokinetic modeling.

Population pharmacokinetic analyses were completed using the Monolix software version 2016R1 (Lixoft SAS, 2016; Antony, France) and the stochastic approximation expectation-maximization (SAEM) (22) for parameter estimation. Simulations were implemented using the Simulx package (http://simulx.webpopix.org/mlxr/) in R (RStudio). Computations were performed using facilities provided by the University of Cape Town Information and Communication Technology Service's High Performance Computing team (http://hpc.uct.ac.za).

Several standard structural models were tested for stavudine triphosphate, as follows: one- and two-phase disposition with first-order disappearance, and first-order appearance, with and without a lag time. Standard plasma pharmacokinetic models were used to model the intracellular data, reflecting the fact that the profile of intracellular pharmacokinetics (PK) is closely related to the plasma exposure (17, 23, 24).

The pharmacokinetic samples collected predose were treated as a separate occasion in the model to allow estimation of both interindividual variability (IIV) and interoccasion variability (IOV). A log-normal distribution was assumed for these random effects, and the correlation between them was investigated at both the IIV and IOV levels. The relative bioavailability was fixed to 1 for a typical patient, and random-effects variability was tested around this reference value. The residual unexplained variability (RUV) was evaluated using a combined additive and proportional model. Censored data below the lower limit of quantification (LLOQ) were handled with the Monolix implementation of the M3 method (25). Briefly, the M3 method maximizes the likelihood of the observation being lower than the LLOQ, instead of fitting the model prediction to the observed concentration, which is the standard procedure used for all noncensored values. Model development was guided by changes in the −2 log-likelihood (−2LL; with drops of more than 6.63 points considered significant at a *P* of <0.01 for the inclusion of 1 additional parameter in the model), precision in parameter estimates (% relative standard error [RSE]), graphical analysis of goodness-of-fit plots, including visual predictive checks (VPCs) (26), and scientific plausibility.

Allometric scaling using fixed coefficients of 3/4 and 1 was applied to all clearance (CL, Q) and volume of distribution (*V*_c_, *V*_p_) parameters, respectively, to account for differences in body size (27). Besides total body weight, fat-free mass (FFM) (28) and body surface area were also tested as alternative descriptors of body size.

After the inclusion of allometric scaling, covariate selection was performed by first narrowing down the search to factors that were either known or physiologically plausible to affect a certain pharmacokinetic parameter. Then, the plots of individual random effects (empirical Bayes estimates) (29) versus covariates were used to screen possibly significant trends in the data. Finally, the candidate covariate effects were tested and included in the model using a stepwise procedure (30) with forward inclusion (*P* < 0.05 based on drop in −2LL) and backward elimination (*P* < 0.01). Additionally, the improvement in goodness-of-fit plots, including VPCs, the reduction in unexplained variability, and the stability of the model parameter estimates, were considered to decide on the retention of the effects in the model.

Children versus adults was used as a categorical covariate to test whether parameter estimates (oral clearance, bioavailability, and the appearance rate constant [*k*_a_]) were different between adults and children in the final model for stavudine triphosphate. Additionally, sex, renal function, and age were tested for significance in the model. Age was tested using a sigmoidal maturation model (31, 32). For renal function, the estimated glomerular filtration rate (eGFR) was calculated using the Cockcroft and Gault equation (33) for adults and the Schwartz equation (34) for children. Since the Schwartz formula has been reported to overpredict eGFR, we accounted for a 14% overprediction for children (35). The eGFR was standardized to a standard male adult weighing 70 kg (FFM, 56.1 kg; body surface area, 1.73 m^2^). The individual values of standardized eGFR were then divided by the median standardized eGFR for the adults in our population to obtain relative renal function (RF). The RF values were used to try to estimate separate values for renal clearance (CLr) and nonrenal clearance (CLnr), as advocated by Holford et al. (32) and shown in the formula below

(1) CL=(CLnr+CLr×RF)×Fsize

where *Fsize* denotes the effect of allometric scaling.

The final model was then used to explore dosing recommendations. First, the exposure in adults receiving the WHO-recommended dose was explored to define the PK target, and then the dosing approach in children was adjusted to achieve the same exposure.

For the *in silico* adult cohort, the covariate values (weight, height, and sex) were obtained from studies conducted at Stellenbosch University (*n* = 2,680). As the current PK study was nested within a concurrent adult randomized clinical trial of stavudine 20 mg twice daily (ClinicalTrials.gov identifier NCT02670772), our model was developed on data from adults receiving a stavudine dose of 20 mg twice daily, but since no evidence exists to suggest nonlinearity in stavudine PK, the model was used to simulate (*n* = 100,000, randomly resampling from the 2,680 individual subject covariate sets) the exposure in adults receiving the current WHO-recommended dose of 30 mg twice daily. The target for AUC, *C*_min_, and *C*_max_ was set to within a 20% difference from the model-predicted median values in this *in silico* cohort of adults.

For the simulations in children, the covariate data set (weight, height, age, and sex) consisted mainly of records from healthy HIV-positive control children attending the Family Infectious Diseases Clinical Research Unit (FAM-CRU) at Stellenbosch University. The initial covariate set (*n* = 793) was resampled to a larger population (*n* = 7,930) by adding random variations (uniform distribution within ±10%) to weight, height, and age. The distribution of the ages (2 to 16 years), weights (interquartile range [IQR], 16 to 25 kg), and FFM (IQR, 13 to 20 kg) were similar to those of the original data set. Based on this set of covariate values, simulations were used (*n* = 100,000, randomly sampling from the population) to explore the exposure in children.

We tested different dosing regimens using weight bands, and doses were rounded off to fall into a stavudine dose range of 0.5 to 0.75 mg/kg twice daily, based on the current available capsules (15, 20, and 30 mg) and liquid formulation (1 mg/ml) strengths.

We refrained from simulating intracellular stavudine concentrations for children under the age of 2 years, since pharmacokinetics is expected to be influenced by maturation in that age range and has not been characterized before.

## Supplementary Material

Supplemental file 1
