# Supplementary material for: Can We Improve Stavudine's Safety Profile in Children? Pharmacokinetics of Intracellular Stavudine Triphosphate with Reduced Dosing
Source: Antimicrob Agents Chemother. 2018 Oct 24;62(11):e00761-18. doi: 10.1128/AAC.00761-18 (PMC6201115; doi:10.1128/AAC.00761-18)
Supplement: Supplemental file 1 [file zac011187548s1.pdf]

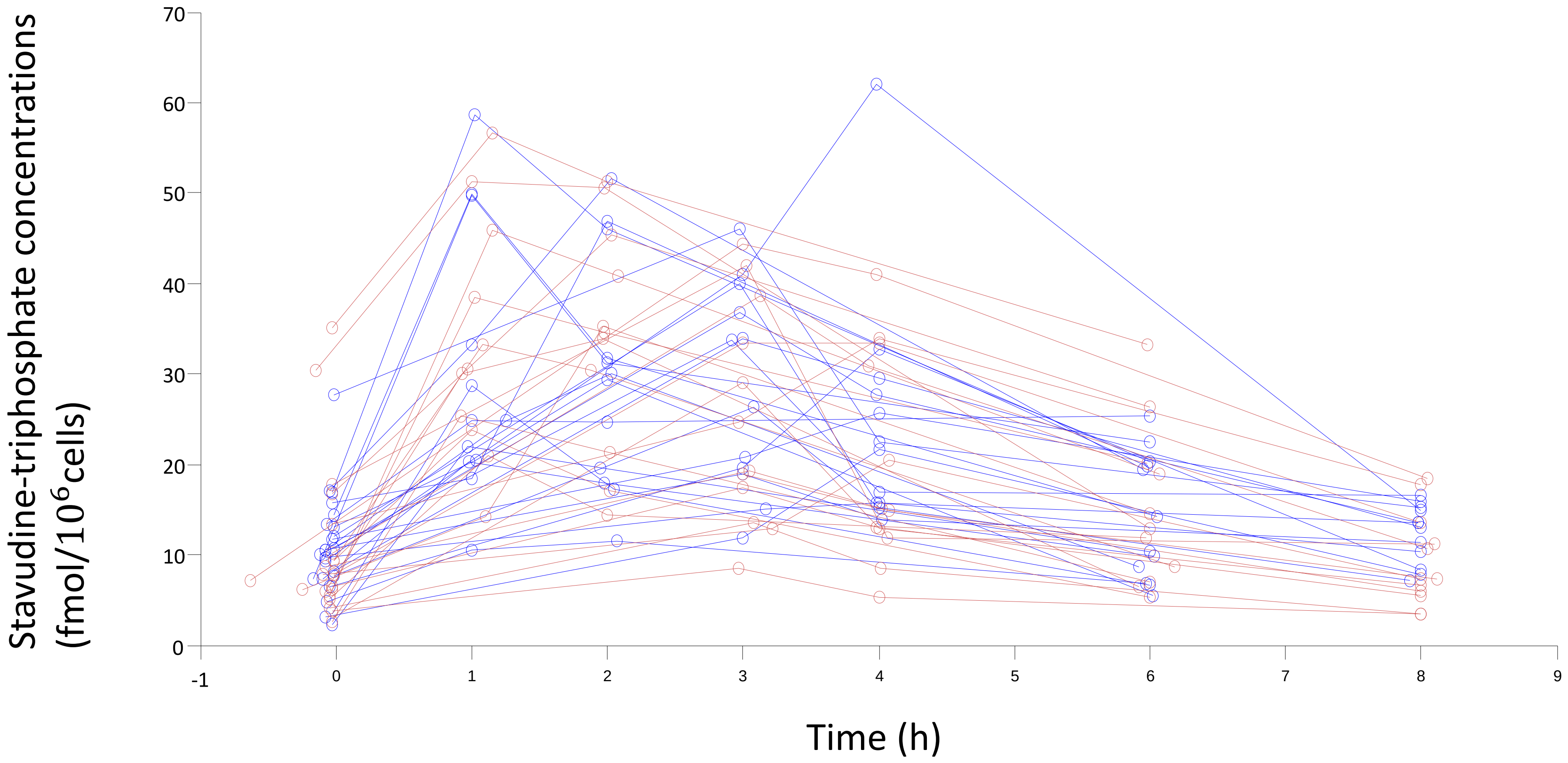

Figure S1: Individual concentration versus time profiles for 23 children (red) and 24 adults (blue) after receiving stavudine at 0.5mg/kg and 20mg twice daily for 7 days, respectively.

Table S1. Secondary pharmacokinetic parameters of intracellular stavudine-triphosphate.

**Pharmacokinetic parameter**	**Adults** (n=24)	**Children** (n=23)
C_{min} (fmol/10^6 cells)	9.3 (6.7-11)	7.0 (5.3-8.8)
C_{max} (fmol/10^6 cells)	32 (25-36)	31 (25-41)
AUC (fmol/10^6 cells · h/L)	232 (154-266)	184 (137-271)
Alpha half-life (h)	1.8 (1.7-1.9)	1.3 (1.2-1.5)
Beta half-life (h)	26 (24-28)	19 (17-21)

Stavudine suspension (1 mg/mL) was used for children and capsules (20 mg) for adults. Median and interquartile ranges are presented.

Table S2. Summary of subject characteristics of the cohort used for *in silico* simulations.

**Characteristics**	**Adults** (n=2680)	**Children** (n=7930)
Age (years)	43 (31-56, 18-96)	7.5 (5.2-8.9, 1.8-16)
No. (%) of males	1174 (44)	3920 (49)
Weight (kg)	67 (56-81, 20-166)	21 (16-25, 6-55)
Fat-free mass (kg)	46 (40-53, 18-62)	17 (13-20, 5-41)
Body Mass Index	24 (20-30, 8-60)	16 (14-18, 9-33)

Median, interquartile range and range are presented unless otherwise stated.
